# Eye fixation during multiple object attention is based on a representation of discrete spatial foci

**DOI:** 10.1038/srep31832

**Published:** 2016-08-26

**Authors:** Meg Fluharty, Ines Jentzsch, Manuel Spitschan, Dhanraj Vishwanath

**Affiliations:** 1 School of Experimental Psychology University of Bristol 12a, Priory Road, Bristol BS8 1TU, United Kingdom; 2 School of Psychology & Neuroscience University of St. Andrews, South Street, St Andrews, Fife KY16 9JP, United Kingdom; 3 Department of Psychology University of Pennsylvania, 3401 Walnut Street, Suite 400A, Philadelphia, PA 19104, USA

## Abstract

We often look at and attend to several objects at once. How the brain determines where to point our eyes when we do this is poorly understood. Here we devised a novel paradigm to discriminate between different models of spatial selection guiding fixation. In contrast to standard static attentional tasks where the eye remains fixed at a predefined location, observers selected their own preferred fixation position while they tracked static targets that were arranged in specific geometric configurations and which changed identity over time. Fixations were best predicted by a representation of discrete spatial foci, not a polygonal grouping, simple 2-foci division of attention or a circular spotlight. Moreover, attentional performance was incompatible with serial selection. Together with previous studies, our findings are compatible with a view that attentional selection and fixation rely on shared spatial representations and suggest a more nuanced definition of overt vs. covert attention.

Visual attention is traditionally assumed to be allocated on the basis of a single movable and scalable spotlight or zoom-lens^1^,^2^, but our subjective impression suggests the capacity for more flexible attentional allocation (for example, when playing a team sport or judging the aesthetic balance of a visual composition). This impression is supported by behavioural studies demonstrating the capacity to selectively attend to multiple non-contiguous locations and delimited objects^3^,^4^,^5^,^6^. Neurophysiological evidence has confirmed these findings showing enhanced neural activation for spatially discrete attended targets in comparison to intervening spatial regions or distractors^7^,^8^,^9^,^10^. However, little is known about how the brain controls the movements of the eyes during such complex attentional allocation.

It is well known that there is a tight coupling between visual spatial selection in attention and the control of eye movements^11^,^12^,^13^,^14^,^15^,^16^, which is reflected in the overlapping neural circuitry underlying these two functions in the frontal eye field, parietal cortex and superior colliculus^17^,^18^,^19^. However, the specific mechanism underlying multi-target spatial attentional selection itself remains a source of active debate. While the idea that selection is based on a single scalable spotlight of attention is discounted by numerous studies^20^, the alternate candidate models, e.g. polygonal grouping, serial switching or discrete spatial foci (See Fig. 1) have remained in contention^8^,^10^,^21^,^22^,^23^,^24^,^25^.

Moreover, the programming and guidance of eye movements may or may not be based on the same representation as attentional selection and monitoring. Since attentional selection (or salience) provides the spatial input to eye movement guidance^26^, fixation could be based on a similar or a coarser representation (e.g., attention selects multiple discrete foci, but fixation is based on a spotlight); Additionally, serial attentional selection or monitoring could yield complex fixation behaviour. Studies of fixation during multiple-object tracking (MOT) have shown that the eye is directed near the centre of the target configuration more often that the targets themselves^27^. However, fine-grained spatial analysis of eye movements to test plausible models of selection (Fig. 1) is challenging with such dynamic stimuli.

In this study we took a novel approach to differentiate among mechanisms of spatial selection, attentional monitoring and fixation guidance during multi-focal attention by measuring eye position and attentional performance during tracking of multiple static alphanumeric targets whose identity rapidly changed over time. Crucially, in contrast to standard static attentional tasks, where fixation is maintained at a predefined location, subjects selected their own preferred fixation location (PFL). Maintaining fixation at an artificially predefined location rarely occurs under natural viewing conditions and could have significant impact on performance^28^ as well as interpretability of the links between attention and eye movements. By allowing fixation to seek a natural gaze position while varying target configuration and numerosity, we aimed to determine (1) if a single stable fixation position, that was not visually pre-specified, could be achieved in a complex attentional task (2) assess what sort of spatial representation would best explain fixation guidance (3) whether this representation was similar to or different from attentional selection and monitoring.

The generic relationship between eye (gaze) position and attentional selection (for saccades and fixation) is based on the primary goal of oculomotor mechanisms, which is to optimize eye position such that target locations/regions are provided equivalent visual resolution^26^,^29^. Since resolution decreases with eccentricity from fixation, the optimal gaze position is the centroid of the selected spatial regions, a prediction supported by the finding that saccade size correlates better with the distance of the centroid of selected targets than the centroid of targets plus intervening distractors^30^. By comparing fixation to multi-target geometries which yield different centroid predictions for the three different models of spatial selection (Fig. 2) we aimed to determine the spatial input guiding fixation. Furthermore, by varying target numerosity to discriminate between parallel and serial monitoring, we aimed to determine if attentional selection, monitoring and fixation are based on similar representations.

## Results: Expriment 1

**Figure 2:**
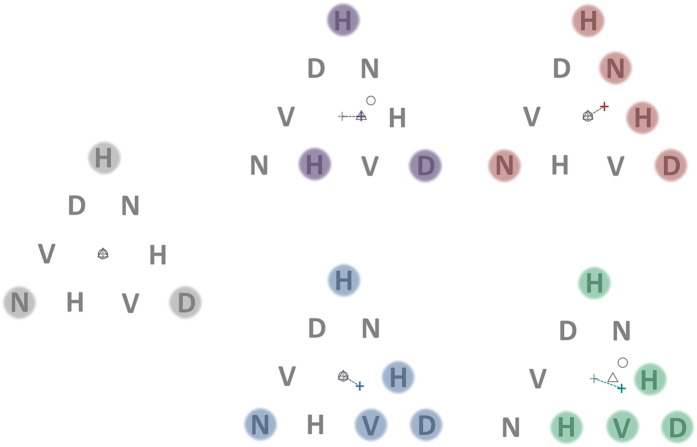
Stimuli for distinguishing among 3 different models of spatial selection underlying fixation. Each stimuli consists of a triangular array of 9 letters that randomly change identity at a rate of 5 hertz. A subject’s eye movements are measured as they monitor a subset of letter locations on the array (indicated by the shaded circles) to detect the occurrence of a briefly presented probe numeral. The subject is required to maintain steady fixation at a location of their choice for the duration of the trial while attending simultaneously to only the target locations. The light grey cross in each panel indicates the centroid of the overall triangular array and initial fixation position at the start of the trial. The coloured cross indicates the geometric centre (centroid) of points marking the centre of the targets. This represents the predicted PFL for multi-foci attention. The outline circle is circumcenter of the outermost target locations and represents the prediction of the circular zoom-lens model. The outline triangle is the centroid of a virtual polygon defined by the target locations and represents the prediction of the virtual-polygon model (note that the centroid of the vertices of the triangle and the centroid of the triangle considered a plane figure (barycentre) are coincident). In the control target configuration shown in the leftmost panel all models of attentional selection predict the same PFL (the centroid of the main triangle). In the top-middle panel, the virtual polygon and multi-foci models predict the same PFL. In the top-right and bottom-middle panels, the zoom lens and virtual polygon models predict the same PFL (centroid of triangle).

**Figure 3:**
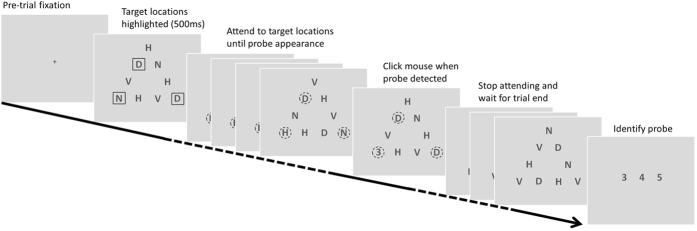
Stimulus sequence during a single trial. A central fixation cued subjects to begin the trial with a mouse click. In the first stimulus frame (500 ms), outline rectangles cued the location of targets to be attended to. After the offset of the outline rectangles, the letters changed identity at a rate of 5 frames per second while the subject attended to the target locations (dotted circles are for illustration only and did not appear in the stimulus). A probe numeral appeared randomly at one of the target locations at a random frame 2000 ms after the offset of the rectangles and 1000 ms before trial end. The subject clicked the mouse upon probe detection, stopped attending to the targets and waited for trial end. At the end of the trial, the subject identified the numeral they detected by selecting from one of three options.

Subjects were required to attend to a pre-defined subset of elements in a triangular array (Fig. 2) and detect the occurrence of a probe numeral at one of these target locations. The stimulus sequence, procedure and instructions are outlined in Fig. 3. In each trial, subjects were instructed to focus their attention simultaneously on the target locations only (initially highlighted by outline boxes) ignoring the other locations in the array. Their instructions were to try to “maintain a single natural gaze position from which you feel you can attend to all and only the target locations simultaneously”. Subjects were asked to click the mouse as quickly as possible when a probe numeral appeared at one of the target locations. No feedback was given and half the trials were catch trials that did not contain a probe (not known to subjects). Every 4^th^ experimental trial was followed by a calibration trial that required sequentially fixating point targets located at each corner of the triangular array.

**Figure 4:**
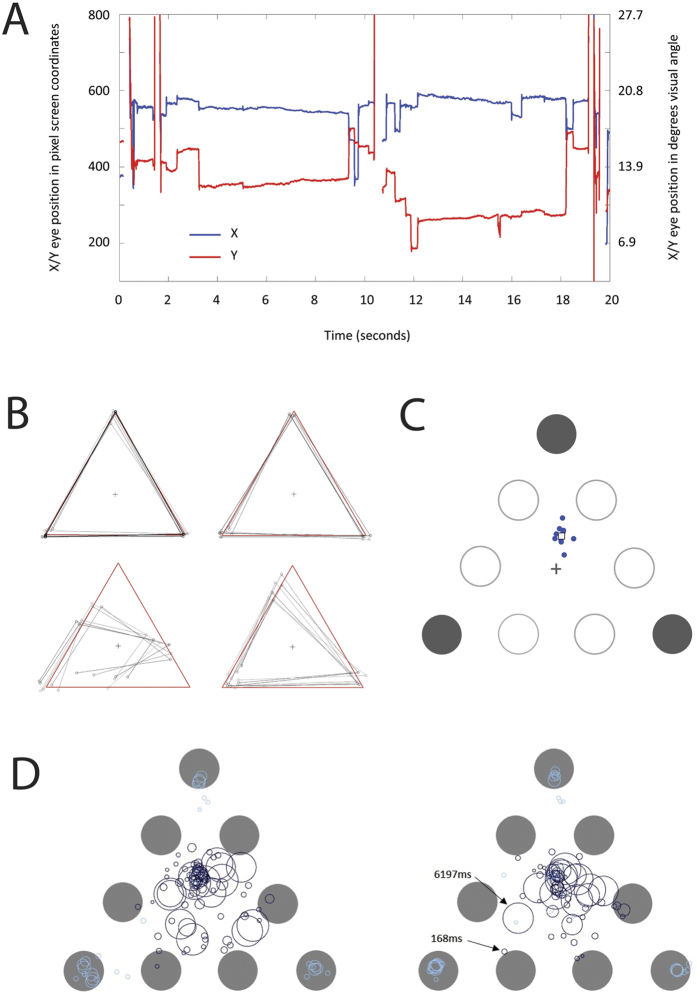
(**A**) Sample eye traces for one subject for two consecutive trials. Large vertical shifts indicate inter trial blinks or loss of tracker lock. (**B**) Mean fixation position from intra-sessional spatial calibration trails. Subjects sequentially fixated a dot that moved from the left corner of the red triangle to the other corners and back again in a counter-clockwise direction (1 second per fixation). The red triangle was not visible during the calibration trial. Black lines indicate vectors between mean fixation locations for calibration trials during each of six experimental sessions. The top two panels are for two representative subjects who were included in the main analysis of fixation data. The bottom panels are for the two subjects that were excluded from the analysis. (**C**) Mean preferred fixation locations (PFLs) for the control target configuration (see Fig. 2, left panel) per subject averaged over all sessions (blue dots). The mean PFL averaged over all subjects is shown by the white square. (**D**) Fixation positions (saccade endpoints) for 12 consecutive trials (including both 3-target and 5-target stimuli) in each of two different testing sessions (left and right panel) for one representative subject. Dark open circles are trial fixations during the attentional task and light blue open circles are fixations during inter-trial calibration trials. The centre of the outline circles are saccade endpoints and their diameter represents the square root of the fixation duration; two representative fixation durations are indicated.

In these intra-sessional calibration trials, 11 out of the 13 subjects tested generated accurate point fixations, with average RMS position error ranging between 0.59°−1.6° (M = 1.07°; SD = 0.34°). Two subjects produced significant average spatial errors of 4.6° and 6.35° respectively. These large errors were confirmed in the visual inspection of the calibration data in comparison to typical performance by other subjects (see Fig. 4B). Since we were interested in measuring absolute fixation location in the main experimental trails, we excluded these two subjects from the fixation analysis as the high levels of noise in a simple sequential fixation task to point targets suggested that their fixation locations for the more demanding attentional task would be unreliable and difficult to interpret.

During the experimental trials, subjects generally showed stable fixations during the main epoch of interest (after the offset of the target highlights and before appearance of the numeric probe). These fixations lasted anywhere from about 2.5 to 6 seconds, indicating that subjects were, in most instances, able to select and maintain a single preferred fixation location during the main attentional phase of the experiment (Fig. 4A,D). There was little or no evidence of systematic serial fixations to targets either before or during the main attentional task phase (Fig. 4D).

To determine preferred fixation locations, we analysed eye position during the first 1200 ms of the attentional task phase for all configurations tested (see methods). For the control 3-target configuration (corners of the triangle; Fig. 2, leftmost panel) all plausible modes of attention allocation (except serial scanning) predicted fixation at the centroid of the equilateral triangle. However, preferred fixation locations (PFLs) for all subjects showed a large upward (vertical) and small rightward (nasal) shift from center (Fig. 4C). Similar upward shift in fixation positions were found in studies of MOT^27^. One possible explanation for the upward bias is that attentional resolution is superior in the lower hemi-field^27^,^31^. Therefore, fixating above the geometric centre could confer more equivalent attentional resolution for targets in the upper and lower hemi-field. The slight rightward shift that we found might be indicative of a stereotypic nasal bias resulting from the fact that we measured only the left eye position. A component of both these shifts could have also arisen from intrinsic biases in correlating measured fixation (based on eye tracker output) and the actual centre of the distribution of attention. We discounted these stereotypic biases by correcting the X and Y component of fixation locations for all experimental target configurations by the horizontal and vertical shifts separately for each subject (Table 1).

**Figure 5:**
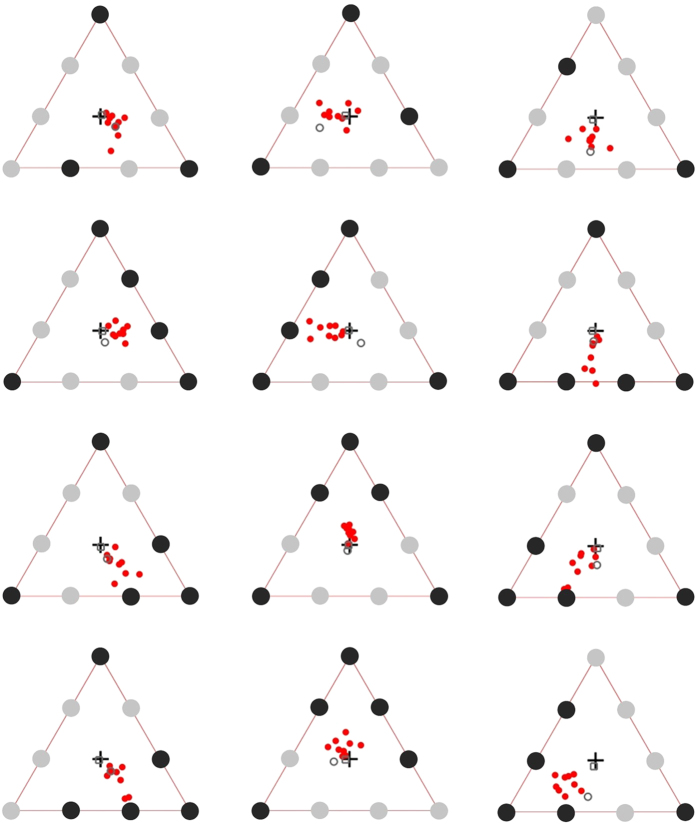
Mean PFLs, per subject, for the four target configurations (rows) and the three orientations (columns) tested for each target configuration. Black disks indicate target locations. The black cross is the centre of the triangle and the location of initial fixation at trial start. Data points (PFLs) represent mean eye fixation position after correcting for offsets observed in the control target configuration (see Fig. 4C). The red dots are the PFLs for each of the 9 typically performing subjects. The outline square and outline circle are mean PFLs for the 2 subjects considered as outliers (see main text). Each configuration in row 1 and row 4 was also tested in the mirror reflected version. PFLs shown are collapsed over the mirror reflected versions. The number of fixations averaged per PFL shown vary among configurations.

The mean preferred fixation location (PFL) during the epoch of interest, per subject, per configuration, after correction for the vertical/horizontal shifts observed in the control stimulus, and collapsing over mirror-reflected versions, is shown in Fig. 5. For 9 subjects (outline circles), the PFLs for each configuration were consistently shifted away from the centre of triangle in the direction of the multi-focal centroids (see Fig. 2). One subject’s PFLs (grey squares in Fig. 5) were consistently located at or close to the triangle centroid location for all target configurations. The triangle centroid is conceivably the default PFL for this subject for all configurations. However, no plausible spatial selection model predicts the same central fixation location for all target configurations, except one where the subject always attended to all the letters in the array regardless of the target configuration. Given that all other subjects showed reliable shifts of the PFL away from triangle centroid for all but the control target configuration, we concluded that this subject most likely misunderstood the instructions, mistakenly assuming that they were required to try to maintain fixation across all trials at the same default fixation location. Our instruction did not explicitly indicate to the subjects that their fixation locations could be different for different configuration, and all trials started with a central fixation cross located in coincidence with the triangle centroid. The last subject (grey outline circle in Fig. 5), produced PFLs that were also shifted away from the triangle centroid in the direction of the multi-foci centroid in most cases, but for which idiosyncrasies were discerned for a few of the configurations. The main analysis therefore focusses on the 9 subjects who showed the same general behaviour for all configurations, with the subject described above considered as an outlier.

**Figure 6:**
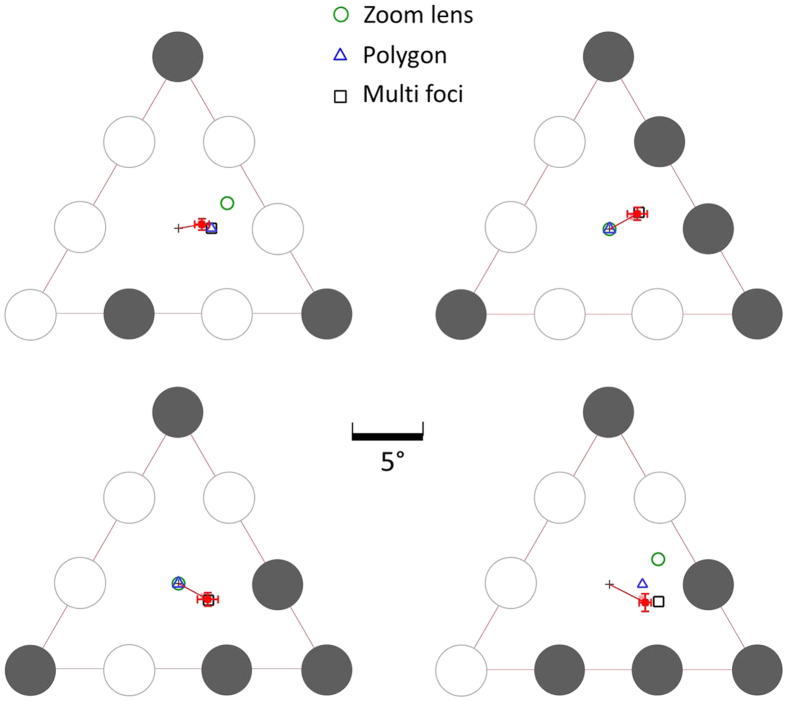
Mean PFLs (red) for the four experimental configurations tested averaged over the 9 typical subjects (see text). Error bars represent 95% confidence intervals for the X, Y coordinates (X and Y data points for each configuration were normally distributed; Shapiro-Wilk test, all p’s > 0.05). The grey cross represents the location of the main triangle centroid and initial fixation location. The outline square, outline triangle and outline circle are (respectively) the multi-foci centroid, the virtual-polygon centroid and the circular zoom-lens centroid. Mean PFLs averaged over all 11 subjects tested is shown as a light red square but is wholly or partially obscured by the red square.

Individual mean fixation locations most likely incorporate orientation-dependent stereotypic and idiosyncratic biases in recorded fixation location, making the interpretation of absolute fixation location for a specific orientation for a specific target configuration difficult. Data for each target configuration (Fig. 2) was therefore collapsed over all orientations tested (Rows in Fig. 5). Mean PFLs for the 9 typical subjects, after collapsing over orientation, are plotted in Fig. 6A as vector deviations from the centre of the triangle array, which was also the location of the initial pre-trial fixation. For all four configurations, the fixation location predicted by the zoom-lens and virtual-polygon models were located outside the 95% confidence intervals of mean PFLs for both X and Y coordinates. In contrast, the PFLs are highly consistent with those predicted by the centroid of discrete multiple foci of attention. In most cases the multi-foci centroids were located within the 95% confidence intervals for both X and Y coordinates; in two cases, the mean PFLs were almost identical to the predicted PFL (Fig. 6A, upper-right, lower-left panels). The predicted PFL in one of the configurations (Fig. 6A, bottom right panel) was located outside the confidence interval for the X coordinate, but only marginally. Our stimulus design was such that including observers with noisy or erratic eye movements, or with different individual biases, predicts a regression of mean PFLs (collapsing over all orientations and subjects) to the centre of the triangular array (initial fixation). Including the two outlier subjects in the overall analysis was consistent with this prediction as there was still a clear and consistent shift in mean PFLs in the vector directions of the multiple spatial foci centroids, but the magnitude of the shift was slightly smaller (light red squares in Fig. 6A; wholly or partially obscured by the red squares).

**Figure 7:**
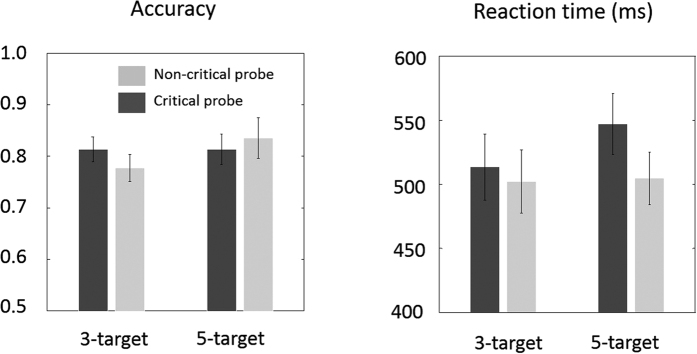
Accuracy (proportion of correct detections) and reaction time data for the 3- and 5-target configurations in the attentional task. Error bars are one standard error of the mean. Dark grey bars are for critical probe trials (probe located at the isolated target) and light grey bars are for non-critical probe trials (probe located at a target in a cluster closer to fixation).

The mean PFLs that we found were consistent with a spatial representation of discrete spatial foci guiding fixation. Since attentional selection provides the spatial input for eye movement guidance, the representation guiding attention could be of a finer resolution than that guiding fixation, but not coarser (e.g. a spotlight). Our fixation result therefore provides the first ocular motor confirmation of previous findings that attentional selection itself must be based on discrete foci^10^. However, is still possible that the attentional monitoring of these locations occurs in a serial fashion (switching model^22^,^23^). In order to test this possibility, we examined the results of the detection task comparing configurations with 3 targets vs. 5 targets. The serial switching model predicts higher reaction times and lower correct detections for the 5-target configurations. We first examined data from the 9 subjects whose mean PFLs are summarized in Fig. 6. Figure 7 shows the proportion correct detections and reaction times for 3- and 5- target trials. The error rate in reporting the identity of the target when subjects correctly detected the presence of a target was low: 3% and 3% respectively for 3- and 5- target configurations. Thus, when a probe was present, and the response was made after probe appearance, identification was nearly perfect. The relatively high probe detection rate and low identification error rate confirms that, in these trials, subjects were effectively allocating attention to the target locations. In non-blank trials, subjects on average reported detection of a probe (mouse button click before trial end and correct identification) in 79.5% of 3-target trials and 82.4% of 5-target trials. False alarms, where subjects indicated detection of a probe numeral before the actual display of a probe or where none was present (blank trials) were 31% for both 3- and 5-target configurations.

As described, the PFLs occurred near, or coincident with, the multi-foci centroids. In the 5-target configurations this meant that fixations were close to a grouping or cluster of target elements (Fig. 6A). Therefore, detectability of probes located on the clusters in the 5-target condition might be heightened simply due to the spatial proximity to fixation position, complicating the interpretation of differences between 3- vs. 5-target detectability. We therefore examined detectability separately for trials where the probe was on the isolated target location (“critical probes”) or on the cluster (“non-critical probes”). Both serial allocation of attention and probe eccentricity would predict poorer detectability for the critical probe in the 5-target configurations. Contrary to the serial allocation hypothesis, we found that correct detection of critical probes in 3-target configurations (81%) were, on average, the same as for critical probes in the 5-target configuration (81%) despite the greater eccentricity of the probe from mean fixation in the latter (see Fig. 6B, top right and bottom panels). Correct detections for 3-target non-critical probes (78%) was numerically lower than for 5-target non-critical probes (84%). However, this difference (opposite in direction to the serial prediction) was not significant. We found no main effect of, or interaction between, target numerosity or probe type on correct detection rate or false alarms, all p’s > 0.10.

In trials for which there were correct detections (Fig. 7, right panel), we found a significant effect of probe type on reaction time (F (1, 8) = 7.57, p = 0.025) and a main effect of target numerosity (F (1, 8) = 12.44, p = 0.008), with faster RTs for 3-target trials (508 ms) than 5-target trials (526 ms). However, although the interaction between target numerosity and probe type was not significant (F (1, 8) = 0.50; p = 0.50), visual inspection of Fig. 7 (right panel) suggests that both main effects were mainly driven by higher RTs for critical 5-target trials (547 ms) than for all other trial types (3-target critical: 514 ms, 3-target non-critical: 502 ms, 5-target non-critical: 505 ms). The magnitude of the target numerosity effect on reaction time (18 ms) was an order of magnitude smaller than predicted based on estimates of the latency for temporal switching (between just two targets) in the literature (125–250 ms)^22^,^24^. Finally, analysing the probe correct-detections and reaction-time data including all 11 subjects for whom eye movement data was analysed produced no significant differences in correct detections or reaction time for 3- vs. 5-target trials, or critical vs. non-critical trials, all p’s > 0.09.

## Results: Experiment 2

Using a different stimulus array we aimed to replicate our fixation localization findings from Experiment 1. Some have argued that previous evidence for multi-focal selection does not specifically rule out hybrid or ad-hoc spatial selection (e.g., attentional window(s) with some unique ad-hoc amorphous shape encompassing targets^23^; though others have argued that such explanations are not parsimonious^22^). The only alternative explanation for the pattern of results in Experiment 1 that could arguably be considered parsimonious is a simple divided attention model where targets are encompassed by two spatial foci (e.g., one encompassing the remote target and one encompassing the cluster of targets). In addition to confirming the results from experiment 1 we aimed to rule out this alternative explanation.

**Figure 8:**
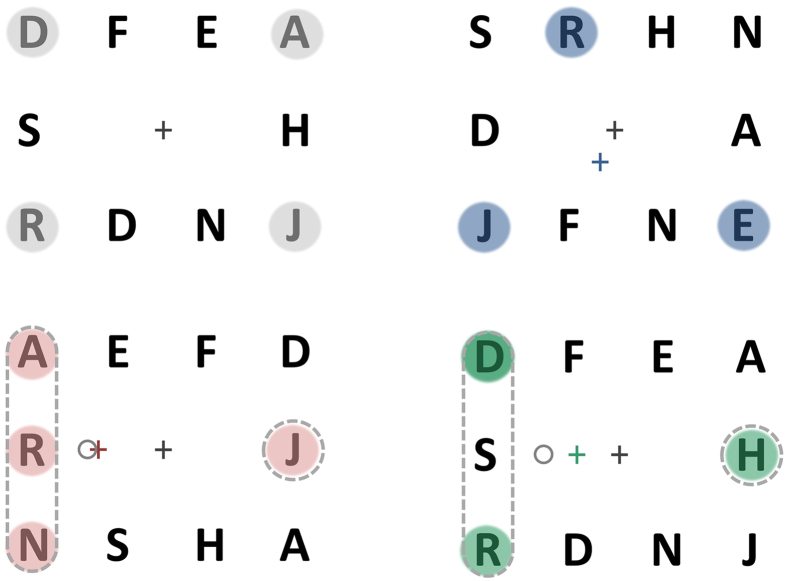
Four target configurations tested in experiment 2. The shaded circles indicate attentional target locations. The top left panel is the control target configuration where all plausible modes of attentional selection predict the same PFL. The two lower panels show the configurations designed to differentiate between multi-focal attention and a rudimentary division of attention into two foci (dotted areas). The dark grey crosses indicate the centre of the overall rectangular array and initial fixation, the coloured crosses indicate the multi-foci centroids for each configuration and the grey circle is the centroid consistent with 2-foci divided attention. In the critical comparison (lower panels), 2-foci divided attention predicts the same PFL for both configurations, while the multi-foci model predicts different PFLs (coloured crosses).

We utilized a different 9 letter rectangular array and constructed 3-target and 4-target configurations (Fig. 8). The critical comparison was between the target configurations shown in two lower panels of Fig. 8, which predict different fixation locations (centroids) for multi-foci selection, but the same fixation position for a simpler 2-foci divided attention model. Each configuration was also tested in their mirrored version, about the vertical axis (for configurations shown in the upper right and lower panels in Fig. 8) and about the horizontal axis (upper right panel in Fig. 8).

**Figure 9:**
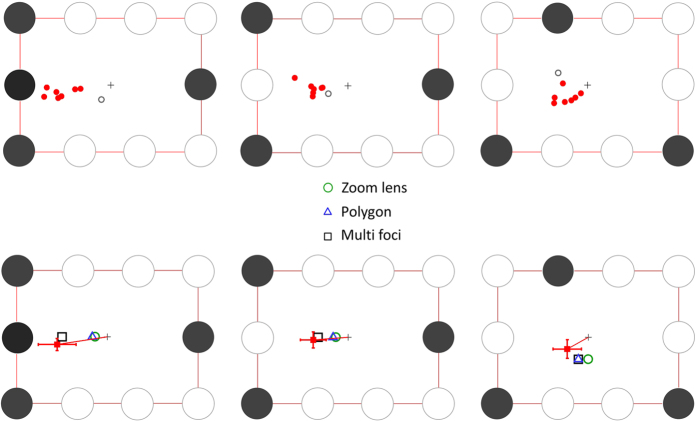
(**A**) Individual mean PFLs (8 subjects) for three test configurations in experiment 2 after correction for stereotypic vertical shifts observed in the control target configuration (Fig. 8, top left panel). In each panel, the black disks indicate the location of the attentional targets within the array. The red dots are the PFLs for the 7 subjects that generally followed the same pattern of fixation as in the previous experiment, while the grey outline circle is the PFLs recorded for the subject who showed an idiosyncratic pattern (see results). (**B**) Mean PFLs averaged over 7 typical subjects (red squares). Error bars are 95% confidence intervals. In the lower panels, the outline squares indicate the multi foci centroids, the green circle is the prediction for the circular zoom lens and the blue triangle for the virtual polygon.

Figure 9 (top panels) shows the mean individual PFLs per subject after correcting for individual vertical/horizontal biases observed in the control target configuration (see Experiment 1). Again, the PFLs were consistently shifted away from initial fixation in the direction of the multi-foci centroids in all three configurations for 7 out of 8 subjects. For these subjects, as predicted by the multi-foci model, the shift in PFL from the initial central fixation was generally greater for the four target configuration (Fig. 9, top left panel) than for the similar 3-target configuration (Fig. 9, top middle panel). The eighth subject displayed an idiosyncratic pattern of fixation, with PFLs clearly not following that observed in the other subjects. Moreover, this subject also indicated at the end of the sessions that they were using a specific strategy to “make the task easier”: they claimed that they visually “blurred” the stimulus by slightly crossing their eyes. We therefore regarded this subject as an outlier and excluded them from the group mean calculation.

The overall mean PFLs (7 subjects) were consistently shifted away from the default central fixation toward the multi-foci centroids (Fig. 9, bottom panels). The vector orientations were not as closely aligned with predictions as observed in Experiment 1. In Experiment 2 we did not test a full range of orientations for each target configuration as in Experiment 1, and the overall array was always aligned with cardinal axes (only mirror reflections were tested; see methods). This may have prevented sufficient rotational averaging-out of idiosyncratic hemi-field and nasal/temporal biases in attentional selection and eye guidance. Notwithstanding this angular mismatch, the main aim of this second experiment was to test whether there was a difference in the X component of the PFLs in the critical comparison between the configurations shown in the left and middle panels in Fig. 9. The statistically significant difference we found between these two configurations (t(6) = 3.57; p = 0.012) was in the direction predicted, and close in magnitude to that predicted by the multi-foci centroids (1.76° versus 1.73°).

While this second experiment was designed mainly to investigate fixation location and not attentional performance (e.g., no blank trials were included), we did examine correct-detection rates and reaction time. Overall, performance measures indicated that probe detection was significantly harder in this experiment given the faster rate at which letters changed identity and the increased number of unique letters in the array. Correct detections occurred on only 57% of trials for the four-target configuration and for 50% of the trials for the 3-target configurations. Moreover, reaction times were significantly higher than in experiment 1 (1102 and 1110 ms respectively for the 3-target and 4-target trials) and variability was much larger, with average standard deviations of about 620 ms. This suggests that parameter choices made the detection task considerably more difficult, and that those chosen for the first experiment were better suited to the analysis of attentional performance.

## Discussion

In the first experiment we found that the majority of subjects were able to select and maintain consistent preferred fixation locations during a demanding multi-target attentional task in the absence of any visual marking for fixation. There with little evidence of systematic serial fixations of targets. Fixation stability was excellent, with many fixations maintained for almost the full duration of the trial (>5 secs). The PFL spatial coordinates were coincident or close to the centroid defined over discrete target locations, not a polygonal grouping of targets, or a circular spotlight. We found little support for serial attentional monitoring in our effects of target numerosity on attentional performance. We replicated the fixation results in Experiment 2, and furthermore, showed that PFLs were inconsistent with simpler 2-foci divided attentional selection. Taken together, our results show that fixational guidance during multiple object attention is based on a representation of discrete foci at relevant locations. The close spatial correspondence between mean PFLs and multi-foci centroids was remarkable given the numerous potential sources of noise and orientation-dependent idiosyncrasies in behaviour and eye-position measurement.

One alternative interpretation of our results is that the PFLs we measured are not indicative of low-level visual demands of providing optimal resolution, but due to higher level factors, such as probe-location probability^32^ or deliberate cognitive biases to fixate closer to the majority (clustering) of targets. We think these explanations are highly unlikely. First, such biases are not statistically optimal since the probe (“reward”) occurred at the isolated target more often than any other location^32^ (see methods). Moreover, there was no detriment to probe-detection accuracy at the isolated location predicted by a deliberate shift of attentional resources away from it (subjects were not operating at ceiling capacity). Finally, it is highly implausible that subject-specific idiosyncratic cognitive biases could have coincidentally yielded such close spatial agreement with centroid predictions, or the specific predicted difference between the critical comparisons in Experiment 2.

Another alternative conjecture is that PFLs were determined by purely oculomotor means rather than input from selection mechanisms, for example, by rapid serial fixations prior to the main fixation epoch. We found little evidence of systematic fixations to targets during the trial (Fig. 4D). Moreover, analysis of fixations for two representative subjects who showed performance closest to the multi-focal centroid prediction) showed that the number of fixations/saccades prior to the main fixation epoch were essentially identical for 3- and 5-target trails and less than the minimum of 3 fixations required to scan even the 3-target trials. Median number of saccades for each subject were 1 and 2 saccades respectively regardless of target numerosity [Means: Subject1, 1.97(3) vs. 1.89(5); Subject2, 2.49(3) vs. 2.48(5)].

Our conclusions ruling out polygonal selection must be qualified by the fact that we used static targets. Dynamic targets (e.g., MOT) may depend on polygonal grouping and show configural effects in either attention or eye movements. For example, during multiple identity tracking of moving targets, both attention and visual working memory have been shown to be affected by whether or not the multiple moving targets retain an overall configuration of a convex polygon^33^,^34^. One way to reconcile these differences is to consider the possibility that MOT recruits higher order attentional mechanisms for dynamic tracking that our task did not require. Fixation may be guided by a more rudimentary low-level representation of discrete targets. For example, Stoermer and colleagues^10^ found accuracy and reaction time affected by target numerosity in MOT, though no similar reduction due to numerosity was found in amplitude differences in SSVEPs between targets and distractors.

While our results are consistent with selection and representation of discrete spatial foci, could these locations have been monitored in serial fashion? Given that we found no decline in performance with increasing number of attention foci as measured by accuracy and false alarm rate and only a small decline in reaction time (of a magnitude much smaller than would be predicted by serial monitoring) our results, consistent with previous studies^9^,^10^, lend further behavioural support for parallel rather than serial monitoring of multiple foci.

Taken together, the fixation and attentional performance results are consistent with the view that fixation localization and attentional selection (at a rudimentary level) may be underwritten by shared spatial representations^35^. They provide converging evidence from a complex behavioural task, that fixation represents the topographically defined stable “equilibrium point” of attentional distribution, reflected in the finding that the population average of the topographic neural activity in SC, modulated by target relevance, determines gaze direction^35^. Our results mirror findings that the centroid of the visually selected multiple targets is also the goal position for saccades^30^. The possibility that both saccades and fixation are driven by a common reference position makes sense. Bringing the line of gaze to the location that will provide equivalent visual and attentional resolution over the selected target(s) at saccade endpoint minimises the need for further saccadic adjustments at fixation, and both types of eye movements can be based on shared neural substrates.

Finally, the results have potential implications for the definition of covert attention. Covert attention is traditionally described as attention to targets away from visual fixation. In our task, though subjects were not restricted to a predefined fixation as in standard attentional tasks, they choose consistent stable fixation locations which never coincided with any of the “overtly” selected targets^27^. This suggests that it may be useful to distinguish between *overt* spatial selection and *covert* or *overt* attentional monitoring; the distinction in the latter case being whether or not fixation is at the natural equilibrium position of overt spatial selection, i.e., whether attention occurs with the relevant natural eye movement^36^. For example, in the classic covert task, fixation is maintained at a point away from the single target, and fixation is “pulled” toward the natural equilibrium point (the target centroid) as evidenced by microsaccade bias toward it^37^,^38^,^39^. This is thought to occur because natural fixation represents the balance point (centroid) of activity of topographically organized neurons in the SC coding attentionally relevant positions, and fixating away from this point (covert attention) alters the balance, triggering microsaccades in the direction of the centroid^40^. Our results suggest that with multi-target attention, overt monitoring entails fixating the multi-foci centroid while covert monitoring entail fixating away from the centroid, predicting saccadic^27^ or microsaccadic bias toward it.

## Methods

### Participants

Participants were undergraduate and graduate students at the University of St. Andrews (18–30 years), who were naïve to the purposes of the study and had normal uncorrected vision (Snellen). All methods and experimental protocols were carried out in accordance with the guidelines of the University Teaching and Research Ethics Board (UTREC) of the University of St. Andrews which approved this study. Written informed consent was obtained from all participants in both experiments.

### Apparatus

The stimuli were presented on a 20 inch/51 cm CRT computer display (Iyama HM204DT) running at a refresh rate of 85 Hz. The display region subtended 40 × 30 cm (1152 × 864 pixels). The display was located at a distance of 57 cm. Stimuli and experimental software were generated by a PC running Matlab with Psychophysics Toolbox^41^ and the Eyelink toolbox^42^. The target elements and background had an average luminance of 3.1 Cd/m^2^ and 13.8 Cd/m^2^ respectively.

### Stimulus design

The stimulus array of 9 letters are shown in Fig. 2. The centre-to-centre letter spacing along the sides of the triangle was about 7° visual angle (70 mm). The letter height was about 2 degrees visual angle (20 mm). Three 5-target configurations and two 3-target configurations were used (Fig. 2). Previous studies have demonstrated that up to 7, and perhaps as many as 9, discrete targets can be attentionally tracked^10^,^43^. One of the 3-target configurations (Fig. 2, top left panel) was the control target configuration for which the PFL is predicted to be the centre of the triangle for all plausible modes of spatial selection. The remaining four target configurations were selected such that the predicted PFL would be different depending on the type of spatial selection guiding fixation (see Fig. 2 and caption).

Each target configuration was tested in all possible orientations (rotations and mirror reflections) within the fixed triangular array yielding a total of 37 unique spatial target configurations (5-target: 24, 3-target: 13) to average out any biases in measured fixation location arising from directional error in tracker measurement, nasal/temporal or upper/lower hemi-field biases in attentional allocation or fixation.

Letter-to-letter spacing was greater than critical flanker spacing specified for radial crowding (half of target eccentricity^44^). Critical spacing for radial crowding is more conservative than for the non-radial flanker layout present in our stimuli. Maintaining fixation at or near the default central position (triangle centre) should therefore not lead to crowding.

Attentional allocation was indexed via the identification (correct detection and reaction time) of a briefly presented probe numeral at one of the target locations. Some of the target configurations had one single isolated target with the other targets clustered together. Randomly assigning the probe numeral to a target location would mean that more probes would occur at the cluster location. In order to control for this, two different probe conditions were created: the “critical probe” was defined as one occurring on the isolated target location(s); the “non-critical probe” was defined as one occurring on one of the more closely grouped targets or cluster. There were an equal number of critical and non-critical probes for each configuration such that the probe numeral appeared at the isolated target location significantly more often than at any other target location. In the control configuration, all three equidistant target locations were considered critical probe locations. Half the trials contained no probe item (blank trials).

The letters displayed in the stimulus array were either D, V, N, or H in Sloan font, the US standard font used in visual acuity testing^45^. The probe numbers were 3, 4 and 5 in Slant font chosen due to its similarity in boldness and structure to Sloan font which does not, to our knowledge, have numerals. The specific letters and numbers were selected on the basis of pilot testing on visual distinguishability of letters and detectability of probes at the stimulus frame rate used (200 ms/5 Hz).

### Procedure

An initial fixation cross indicated to the subject that the trial could be started with a mouse click (Fig. 3). At trial initiation the triangular array was presented with the targets to be attended outlined by black rectangles. After 500 ms the boxes disappeared and each letter in the array started to randomly change identity at a rate of 5 Hz. There were 30 display frames in any given trial, following the initial 500 ms (frame zero), resulting in a total trial duration of 6500 ms. The probe numeral appeared at one of the target locations for a single frame duration (200 ms) randomly selected between frame 10 (2000 ms after the letters started to change) and frame 25 (5000 ms after the letters started to change). No probe ever appeared after frame 25, i.e., 1000 ms before the end of the trial. The subject’s reaction time for detecting the onset of the probe was recorded. At the end of the trail, a response screen with a choice of three probe numerals (‘3’, ‘4’ or ‘5’) was shown, with which the subject verified the identity of the probe that they detected (33% chance rate). The monitor remained blank for 900 ms after response and before a new trial start was prompted by the presentation of the fixation cross (Fig. 3). The experiment consisted of 228 trials (presented in 6 sessions of 38 trials each), which included 84 blank, 78 critical and 66 noncritical probe trials. The greater number of critical trials in total reflects the fact that in the control 3-target configuration, the target locations were equidistant and therefore all probe locations were defined as critical.

### Eye tracking

An Eyelink 1000 video-based infrared eye tracker, detected the position of the left eye pupil at a sampling rate of 1000 Hz with a spatial accuracy of 0.25°−0.5° and a spatial resolution of 0.01° (RMS). The subject’s head was stabilized on a chin/head rest and tracker was mounted overhead on the proprietary Tower Mount such that the observer viewed the display through a half silvered mirror.

Subjects were individually calibrated at the start of each session using the Eyelink proprietary calibration and validation function until average error between calibration positions and validation positions was less than 0.5°. In cases where we were unable to successfully calibrate a subject based on this criteria after a few attempts (for example, due to eyelid or eyelash issues, lack of eye stability, etc.) subjects were given partial payment and not tested any further.

Experimental calibration trials were presented after every forth attentional tracking trial. In these trials subjects fixated a single dot that started at the left corner location of the stimulus triangular array (triangle not shown during calibration) and moved successively to the other corners of the triangle in a counter-clockwise direction (1000 ms at each location) before returning to the left corner.

### Eye movement analyses

#### Determination of fixation position

Per trial, we were interested in the fixation location after the disappearance of the target markers but before the possible occurrence of a probe and/or a detection mouse response from the subject. In all trials, the probe appeared on a random selected frame 2000 ms after the disappearance of the target markers and 1000 ms before the end of the trial (between frame 11 and 25). For this reason, we defined the PFL as the average eye position during the 1200 ms epoch that started 400 ms after the disappearance of the target markers and the start of the rapid change in letter identities (frame 3–9). Fixation location was sampled by the experimental program at the stimulus frame rate (every 200 ms) by querying X-Y position from the tracker computer. Real-time and offline inspection of the full resolution X-Y eye position during the duration of the trial confirmed that subjects were, in the vast majority of trials, maintaining a stable single gaze position during the time from the disappearance of the target markers and the subject response or end of trial (Fig. 4A).

#### Correction of fixation position

The X and Y components of fixation locations for all experimental target configurations were corrected by the horizontal and vertical shifts observed in the control configuration (targets at three corners of the triangle) separately for each subject. This type of correction, based on a fiducial control stimulus, has been employed in previous studies that have examined saccadic localization of spatially extended targets, and allows for removal of eye tracker and subject specific idiosyncratic upper/lower hemi-field or nasal/temporal biases before analysis of the effect of experimental parameters of interest^46^,^47^.

### Participants, Apparatus, Stimuli and Procedure: Experiment 2

3 subjects from the previous experiment (whose performance closely matched the mean shifts observed in experiment 1) and 5 new subjects were tested. Display parameters and procedure were the similar to the first experiment except as follows. The average luminance of the targets and background was 4.2 Cd/m^2^ and 22.5 Cd/m^2^ respectively. The frame rate was doubled to 10 Hz to bring the value closer to standard RSVP. We increased the number of possible letters in the array (ADEFHJNRS). We added a 1000 ms lag between the disappearance of the outline rectangles indicating target positions, and the start of the RSVP change in letter identity, so that subjects would have more time to select the targets and adjust their gaze position before the start of the visual transients. Each configuration shown in Fig. 7 was sampled 20 times (including mirrored versions, where applicable) and no blank trials were included.

## Additional Information

**How to cite this article**: Fluharty, M. *et al*. Eye fixation during multiple object attention is based on a representation of discrete spatial foci. *Sci. Rep.* **6**, 31832; doi: 10.1038/srep31832 (2016).

## Figures and Tables

**Figure 1:**
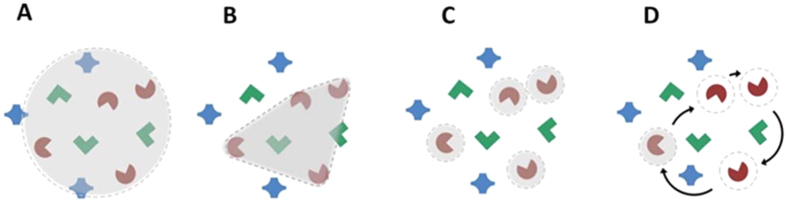
Illustration of four possible mechanisms underlying the capacity to select and monitor multiple targets amid distractors. (**A**) The traditional ‘spotlight’ model: attentional selection is over a circular or elliptical region encompassing both the relevant targets and neighbouring extraneous items (**B**) Virtual polygon: attention is maintained by tracking a virtual polygonal shape whose vertices are defined by the target items. (**C**) Parallel selection of discrete multiple foci of attention. (**D**) Serial monitoring: attentional focus rapidly switches from target to target. It is plausible that a form of covert serial monitoring might combine with a parallel spatial selection such as that shown in **B** or **C**. Note that while the target items in the figure are defined by a feature conjunction (colour and shape), the experiments described in this paper rely on endogenous selection, where there is no visual feature distinguishing target and distractor during attentional tracking.

**Table 1:** Horizontal and vertical correction factors (arcdeg) derived from PFLs for the control stimulus.

Subject	X correction	Y correction
1	−0.4	−2.5
2	−1.5	−2.7
3	−0.3	−3.5
4	−0.6	−3.2
5	−0.4	−2.4
6	−0.7	−1.2
7	−0.6	−3.4
8	−0.5	−4.5
9	0.1	−2.7
10	0.0	−3.1
11	−0.6	−2.6
Mean	−0.5	−2.9
Standard deviation	0.4	0.8
