# Erratum: Eye fixation during multiple object attention is based on a representation of discrete spatial foci

**DOI:** 10.1038/srep46777

**Published:** 2017-05-04

**Authors:** Meg Fluharty, Ines Jentzsch, Manuel Spitschan, Dhanraj Vishwanath

Scientific Reports 6: Article number: 3183210.1038/srep31832; published online: 08 26 2016; updated: 05 04 2017

In the original version of this Article, Affiliations 1 and 2 were not listed in the correct order. The correct affiliations are listed below:

Affiliation 1

School of Psychology & Neuroscience University of St. Andrews, South Street, St Andrews, Fife KY16 9JP, United Kingdom.

Affiliation 2

School of Experimental Psychology University of Bristol 12a, Priory Road, Bristol BS8 1TU, United Kingdom.

This error has now been corrected in the PDF and HTML versions of the Article.

